# Subicular activation preceding hippocampal ripples *in vitro*

**DOI:** 10.1038/srep02696

**Published:** 2013-09-18

**Authors:** Hiroaki Norimoto, Nobuyoshi Matsumoto, Takeyuki Miyawaki, Norio Matsuki, Yuji Ikegaya

**Affiliations:** 1 Laboratory of Chemical Pharmacology, Graduate School of Pharmaceutical Sciences, The University of Tokyo, Bunkyo-ku, Tokyo, Japan; 2 Center for Information and Neural Networks, Suita City, Osaka, Japan; 3 These authors contributed equally to this work.

## Abstract

Sharp wave-ripple complexes (SW-Rs), a transient form of high-frequency field oscillations observed in the hippocampus, are thought to mediate memory consolidation. They are initiated mainly in hippocampal CA3 area and propagate to the entorhinal cortex through the subiculum; however, little is known about how SW-Rs are initiated and propagate. Here, we used functional multineuronal calcium imaging to monitor SW-R-relevant neuronal activity from the subiculum at single-cell resolution. An unexpected finding was that a subset of subicular neurons was activated immediately before hippocampal SW-Rs. The SW-R-preceding activity was not abolished by surgical lesion of the CA1-to-subiculum projection, and thus, it probably arose from entorhinal inputs. Therefore, SW-Rs are likely to be triggered by entorhinal-to-CA3/CA1 inputs. Moreover, the subiculum is not merely a passive intermediate region that SW-Rs pass through, but rather, it seems to contribute to an active modification of neural information related to SW-Rs.

The hippocampal formation, which is composed of the hippocampus, the subiculum, the presubiculum, the parasubiculum and the entorhinal cortex, has a crucial role for learning and memory. Sharp wave-ripple complexes (SW-Rs) occur in the hippocampus^1^,^2^ and have been implicated in memory consolidation^3^. Hippocampal SW-Rs are triggered by a population burst of CA3 pyramidal neurons^4^ and propagate to the hippocampal formation, and therefore, they are universally observed in the hippocampal CA1 region, the subiculum, the presubiculum and the entorhinal cortex *in vivo*^5^. The subiculum, which is located between the hippocampal CA1 region and the presubiculum, is the major target of CA1 output^6^ and also receives direct synaptic inputs from entorhinal cortical and subcortical regions^7^,^8^,^9^. Thus, it may integrate information of the hippocampus and the entorhinal cortex; however, physiological evidence for this notion is still sparse, and the spatiotemporal neuronal activity in the subiculum during SW-Rs remains unclear.

Because *in vivo* studies cannot show a spatial pattern of neuronal activities, *in vitro* evaluations are necessary to reveal the spatiotemporal patterns of SW-R activity and their cellular mechanisms^10^,^11^,^12^. Here, we combined local field potential (LFP) recording with a functional multineuronal calcium imaging (fMCI) technique^13^ and monitored SW-R-relevant spiking of subicular neuron activity with cellular resolution in acute slices of the mouse hippocampal formation. We unexpectedly found that a fraction of subicular activation occurred before hippocampal SW-R events.

## Results

**Figure 1:**
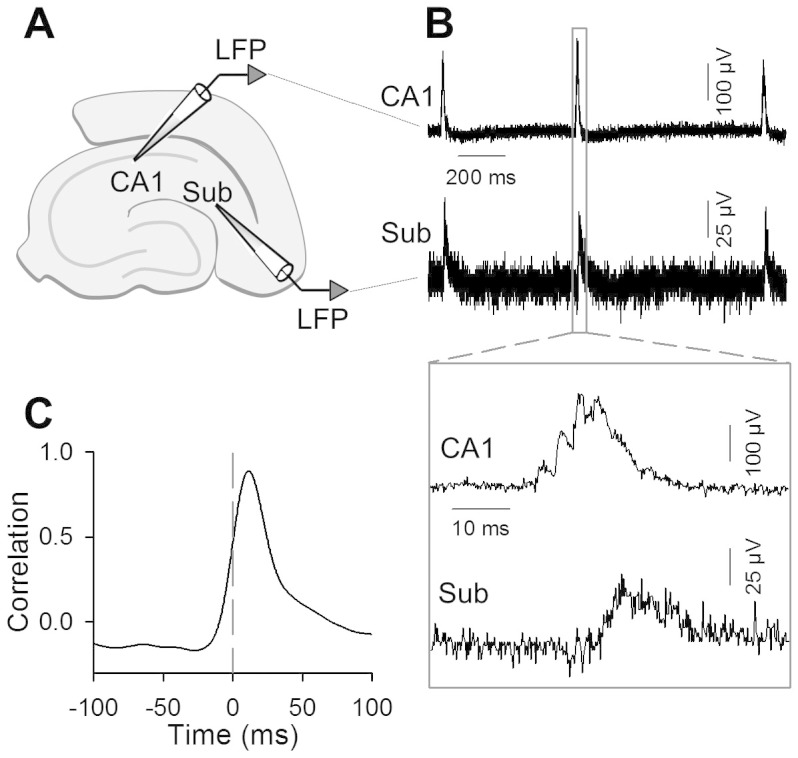
Subicular SW-Rs occur after CA1 SW-Rs. (A) LFPs were recorded from the hippocampal CA1 region and the subiculum. (B) Representative raw LFP traces in the CA1 (top) and the subiculum (Sub, bottom). The boxed parts were time-magnified in the inset. (C) Representative cross-correlogram of band-passed (2–30 Hz) LFP traces recorded from the CA1 and the subiculum.

We recorded LFPs simultaneously from the hippocampal CA1 and subiculum and investigated the temporal correlation of SW-Rs in those regions (Fig. 1A). In all slices tested, SW-Rs occurred spontaneously. The frequency of SW-R events was 0.51 ± 0.11 Hz (mean ± SEM of 7 slices from 4 mice). SW-Rs were observed in both the CA1 region and the subiculum, but they had a small time lag (Fig. 1B). LFP cross-correlogram reveals that SW-Rs in CA1 preceded those in the subiculum (Fig. 1C) and that the mean peak offset was 13.0 ± 2.9 ms (mean ± SEM of 7 slices from 4 mice).

**Figure 2:**
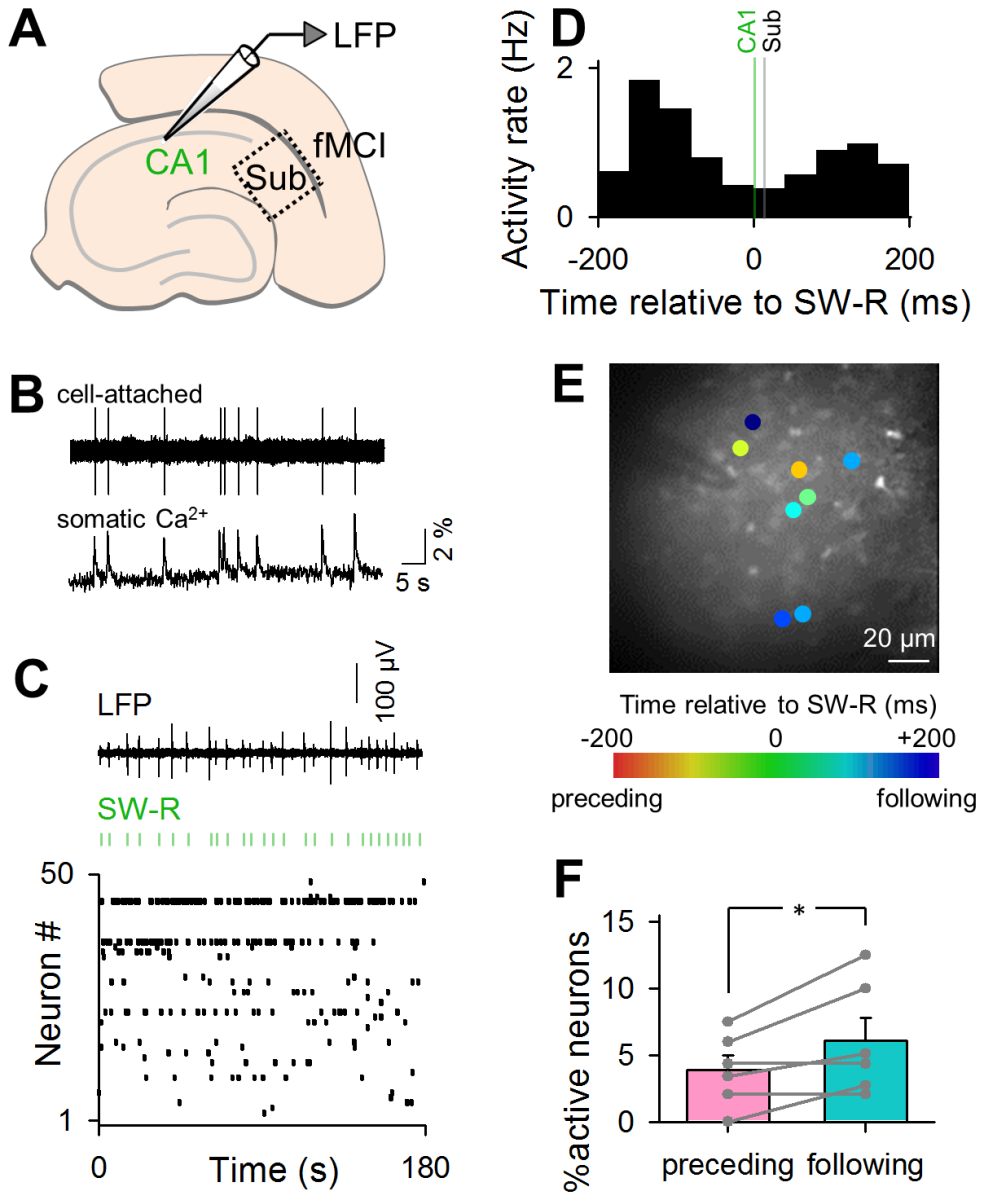
Optical imaging of subicular neuronal activity during SW-Rs in intact slices. (A) fMCI from the subiculum was conducted simultaneously with LFP recording from the CA1 region. (B) Simultaneous cell-attached recording and calcium imaging. (C) Representative band-filtered (2–30 Hz) LFP trace (top) and raster plot of calcium transients of subicular neurons (bottom). Green lines indicate the timings of SW-Rs detected in CA1 LFPs. Each dot in the raster plot indicates a single calcium transient of the corresponding neuron. (D) Peri-SW-R time histogram of calcium transients recorded in 258 subicular neurons. The green line indicates the timings of SW-Rs in CA1, whereas the black line indicates those in the subiculum (sub). (E) A confocal image of OGB1-loaded subicular neurons is merged with neurons exhibiting SW-R-related activities in a pseudocoloured scale that indicates the mean calcium activity timings relative to the SW-R timings. (F) Percentages of SW-R preceding neurons to the total active neurons are significantly higher than that of SW-R following neurons. Error bars represent SEM. *P = 0.023, paired *t*-test of 6 slices.

Spiking activity was optically captured from subicular neurons using fMCI, while LFPs were recorded from CA1 (Fig. 2A). fMCI detects action potentials through action potential-evoked transient calcium elevations in the cell bodies of individual neurons^13^; simultaneous recording of cell-attached recordings and fMCI from the same cells revealed that spikes were tightly associated with individual somatic ΔF/F transients (Fig. 2B). Using a fast-scanning Nipkow confocal microscope, calcium activity was monitored at 50 frames per second from an area of approximately 160 × 150 μm^2^, which included an average of 43 ± 5 neurons (mean ± SEM of 6 slices from 3 mice; ranging from 23 to 59 neurons). A representative raster plot and the corresponding LFP trace are shown in Fig. 2C. Among a total of 50 neurons, 28 neurons (56%) exhibited at least one calcium transient during our recording period of 3 min. The frequency of calcium transients in the active neurons was 0.051 ± 0.020 Hz, on average, ranging from 0.0056 Hz to 0.51 Hz. Some calcium transients were time-locked to SW-Rs; 10.0 ± 2.8% of the calcium spikes occurred within 200 ms relative to the SW-R peak time (mean ± SEM of 6 slices). These SW-R-relevant spikes were observed in 29% active neurons. To classify the firing type of these neurons, we conducted cell-attached patch-clamp recordings and found that 10 out of 12 active neurons were burst-spiking cells. We pooled SW-R-relevant spikes recorded from all 6 slices and plotted the peri-SW-R time histogram in which the timings of individual spikes were aligned to individual SW-R times to examine the net change in the spike frequency relative to SW-R events. The histogram showed two distribution peaks before and after SW-Rs, indicating that the subiculum was biphasically activated before and after CA1 SW-Rs, whereas it was relatively silent during CA1 SW-Rs (Fig. 2D).

We next investigated spike behaviours of individual neurons. Neurons were basically separable into two types, *i.e.*, neurons that fired spikes preceding and following SW-Rs (Fig. 2E), whereas only a few neurons (15.1% of SW-R-relevant neurons) were biphasically activated. As a whole, ‘following' neurons were significantly more in number than ‘preceding' neurons (Fig. 2F; paired *t*-test, *P* = 0.023, *t*_5_ = 3.25, *n* = 6 slices). The fact that there existed subicular neurons that were activated before hippocampal SW-Rs suggests that the subiculum is not merely an intermediate region that relays hippocampal SW-Rs to the entorhinal cortex.

**Figure 3:**
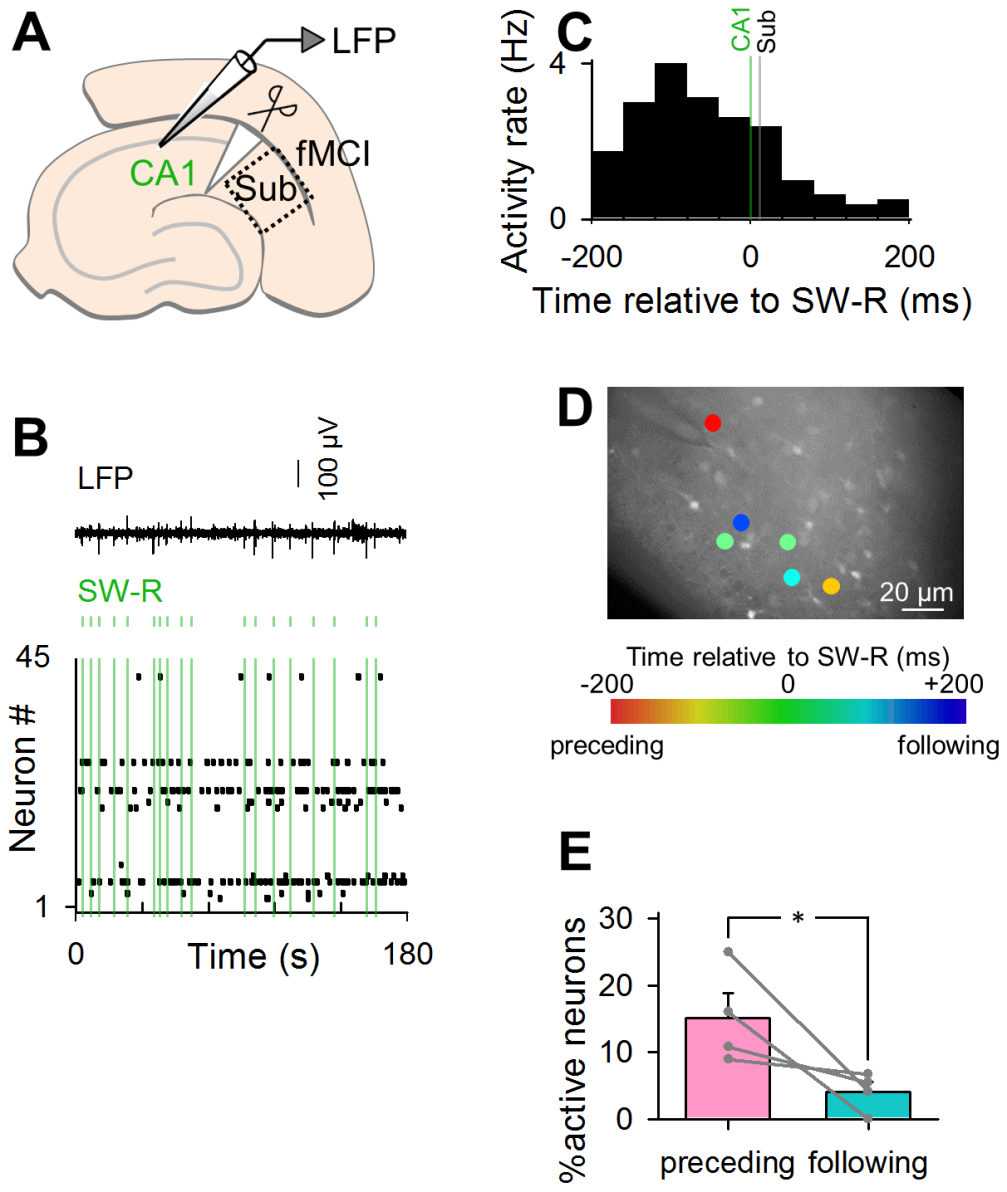
Optical imaging of subicular neuronal activity during SW-Rs in slices that received a surgical incision between the CA1 and the subiculum. (A) fMCI from the subiculum was conducted simultaneously with LFP recording from the CA1 region. (B) Representative band-filtered (2–30 Hz) LFP trace (top) and raster plot of calcium transients of subicular neurons (bottom). Green lines indicate the timings of SW-Rs detected in CA1 LFPs. Each dot in the raster plot indicates a single calcium transient of the corresponding neuron. (C) Peri-SW-R time histogram of calcium transients in 131 subicular neurons. The green line indicates the timing of SW-R in CA1. The black line indicates in subiculum. (D) A confocal image of OGB1-loaded subicular neurons is merged with neurons exhibiting SW-R-related activities in a pseudocolored scale that indicates the mean calcium activity timings relative to the SW-R timings. E. Percentages of SW-R preceding neurons to the total active neurons are significantly lower than that of SW-R following neurons. Error bars represent SEM. *P = 0.042, paired *t*-test of 4 slices.

Subicular neurons received excitatory synaptic afferents mainly from the CA1 region and the layer III of the entorhinal cortex. To investigate how the activation pattern of subicular neurons is shaped by CA1 inputs, we carefully cut the anatomical border between the CA1 and the subiculum using a surgical mini-knife (Fig. 3A). A representative raster plot is shown in Fig. 3B. SW-Rs in the subiculum were abolished after the lesion, although the CA1 region still exhibited SW-Rs at a frequency of 0.28 ± 0.051 Hz, which frequency did not differ from that in intact slices (Student's *t*-test, *P* = 0.15, *t*_8_ = 1.59). Subicular activities were compared relative to the timings of CA1 SW-Rs. In the entire datasets, 19.2 ± 3.3% of the total calcium activities (mean ± SEM of 4 slices) occurred from −200 ms to 200 ms relative to CA1 SW-Rs, but we did not find a peak of the spike distribution after SW-Rs in the time histogram in CA1-disconnected subiculum (Fig. 3C,D). Indeed, ‘preceding' neurons were significantly more in number than ‘following' neurons (Fig. 3E; paired *t*-test, *P* = 0.042, *t*_3_ = 3.25, *n* = 4 slices). Therefore, the ‘following' neurons were likely activated by CA1 afferents.

## Discussion

We found that the subiculum includes two types of neurons in terms of their activity timings relative to SW-Rs; some subicular neurons fired action potentials prior to SW-Rs, whereas others did after SW-Rs. The latter was abolished by a surgical ablation of CA1-to-subiculum projection. Given that the subiculum mainly receives synaptic inputs from the CA1 region and the entorhinal cortex, we speculate that SW-R-preceding activity is triggered by the entorhinal cortex, whereas SW-R-following activity is propagated from the CA1 region. A strategy to more firmly confirm this idea is to examine the effect of the surgical lesion of entorhinal afferents on SW-R activity, but the anatomical feature did not allow us to dissect entorhinal-to-subicular axons; note that these axonal fibers are intermingled with entorhinal-to-hippocampal axons.

An alternative possibility is that SW-R-preceding activity arises from the perirhinal and prefrontal cortices, both of which also provide direct synaptic inputs to the subiculum, but to a much less extent than the entorhinal cortex. Moreover, several subcortical structures, including the ventral premammillary nucleus, the medial septum/nucleus of the diagonal band, and the anteromedial and anteroventral nuclei of the thalamus, are also the sources of subicular afferents^7^,^8^,^9^. However, these subcortical regions were not preserved in our acute slice preparation, and their contributions to the SW-R genesis, if any, are minimal.

Intuitively, the fact that the activities of the preceding and following neuron populations had long offsets of around ±150 ms relative to SW-Rs seemed to be surprising. We have no specific idea to explain the long delays; however, it should be noted that SWs in LFPs mainly represent synaptic inputs, whereas calcium activities represent action potentials, rather than synaptic inputs. Therefore, the delays may arise from a time lag between synaptic inputs and the resultant action potentials.

In conclusion, we discovered that SW-R-related neurons in the subiculum can be classified into SW-R-preceding and SW-R-following neurons. Therefore, the subiculum is likely to contribute both to the generation and propagation of SW-Rs, although it was previously believed to simply relay hippocampal SW-Rs to the entorhinal cortex. Moreover, neurons that were biphasically activated before and after SW-Rs, even though they were numerically less dominant, existed in the subiculum, suggesting that the subiculum actively modifies SW-Rs from the hippocampus. Finally, fMCI detects only suprathreshold activity of neurons, and we could not disclose the subthreshold membrane voltage responses during SW-Rs. Further investigations using voltage-sensitive imaging or optically targeted patch-clamp recording will address more detailed dynamics of neural interaction in the subiculum.

## Methods

### Animal ethics

Experiments were performed with the approval of the animal experiment ethics committee at the University of Tokyo (approval number: 24–8) and according to the University of Tokyo guidelines for the care and use of laboratory animals.

### Slice preparation

Acute slices were prepared from the hippocampal formation of 3-to-4-wk-old male ICR mice. Briefly, a posterior brain block was cut into 400-μm-thick oblique slices at an angle of 12.7° in the fronto-occipital axis using a vibratome in ice-cold oxygenated cutting solution consisting of (in mM) 222.1 sucrose, 27 NaHCO_3_, 1.4 NaH_2_PO_4_, 2.5 KCl, 1 CaCl_2_, 7 MgSO_4_, 0.5 ascorbic acids^14^. Slices were transferred in oxygenated artificial cerebrospinal fluid (aCSF) containing (in mM) 127 NaCl, 3.5 KCl, 1.24 KH_2_PO_4_, 1.2 MgSO_4_, 2.0 CaCl_2_, 26 NaHCO_3_, and 10 d-glucose at 35°C and were allowed to recover for at least 1.5 h.

### Electrophysiological recording

Experiments were performed in a submerge chamber perfused at 8–10 ml/min with oxygenated aCSF at 33–36°C. LFPs were recorded from CA1 stratum pyramidale or the superficial layer of the subiculum using borosilicate glass pipettes (1–2 MΩ) filled with aCSF. Signals were amplified by MultiClamp 700B (Molecular Devices, Union City, CA, USA), digitized at 10,000 Hz and filtered with a band of 1–2,000 Hz by pCLAMP 10 (Molecular Devices). Offline analysis was conducted using custom-made MATLAB routines (MathWorks, Natick, MA, USA). To detect SW-Rs, LFP traces were band-pass filtered at 2–30 Hz and thresholded at 4 times above the SD of the baseline noise. Cell-attached patch-clamp recordings were obtained from subicular neurons with an Axopatch 700B amplifier (Molecular Devices, Union City, CA). Borosilicate glass pipettes (4–7 MΩ) were filled with aCSF. Signals were low-pass filtered at 1–2 kHz, digitized at 20 kHz and analysed with pCLAMP 10.2 software (Molecular Devices).

### Optical recording

Functional multineuron calcium imaging (fMCI) was conducted using acute slices loaded locally with Oregon Green BAPTA1-AM (OGB1)^13^. Fluorophores were excited at 488 nm and visualized using a 507-nm long-pass emission filter. Videos were at 50 frames/s using a 16× objective (CFI75LWD16xW, Nikon, Tokyo, Japan), a Nipkow-disk confocal microscope (CSU-X1; Yokogawa Electric, Tokyo, Japan), and a cooled EM-CCD camera (iXon DU897, Andor, Belfast, UK). The fluorescence change was measured as (*F_t_*− *F_0_*)/*F_0_*, where the *F_t_* is the fluorescence intensity at a given time point; *F_0_* is the baseline. Spike-elicited calcium transients were semi-automatically detected with custom-written Visual Basic software and visually inspected^15^.

## Author Contributions

H.N., N.M., Y.I. designed research; H.N., T.M. performed experiments; H.N., N.M. analysed data; H.N., N.M., T.M. Y.I. discussed results; H.N., N.M., Y.I. wrote the manuscript with help from all authors.
